# National and Subnational Cancer Incidence for 22 Cancer Groups, 2000 to 2016: A Study Based on Cancer Registration Data of Iran

**DOI:** 10.1155/2021/6676666

**Published:** 2021-07-12

**Authors:** Javad Khanali, Ali-Asghar Kolahi

**Affiliations:** Social Determinants of Health Research Center, Shahid Beheshti University of Medical Sciences, Tehran, Iran

## Abstract

**Background:**

Cancer is an increasing public health concern, and detailed knowledge of the cancer incidence is required for developing effective cancer control plans. The objective of this study is to present the cancer incidence of 22 cancer groups in Iran and all 31 provinces of the country from 2000 to 2016, for both sexes across different age groups.

**Method:**

To study the national and provincial cancer incidence in Iran, we extracted data from the Cancer Project, which collects the Iranian cancer registry data and visualizes it in the VIZIT data visualization system. The methodology and statistical analysis that is used in this study follow the cancer project study protocol. Joinpoint analysis was performed to calculate the average annual percent change of the crude rates and age-standardized rates from 2000 to 2016.

**Results:**

Cancer incidence was 126,982 patients in 2016, and the crude rate (CR) of cancer in both sexes and all ages was 155 per 100,000 people. Cancer incidence approximately doubled between 2000 and 2016; however, the age-standardized rate (ASR) had a less drastic increase. The most incident cancers in 2016 were breast, skin, and colorectal cancers; however, the ranking of cancer groups by incidence was different in different age and sex groups and provinces. Some cancers exhibited a unique distribution pattern in the country with high-incidence local areas. *Discussion*. The study showed that cancer incidence, crude rate, and age-standardized rate (ASR) in Iran had increased in 2000-2016 with vast heterogeneity by cancer type, province, and sex. Moreover, it was shown that the crude rate of cancer in Iran was much less than the global cancer crude rate. Providing such data helps to allocate resources and develop effective national cancer control plans appropriately.

## 1. Introduction

Cancer is an increasing public health concern [1]. Cancer incidence was 17.2 million patients worldwide in 2016, which shows a 28% rise since 2006. Cancer also imposed 213.2 million disability-adjusted life years (DALYs) on public health in the same year [2]. Moreover, it is estimated that global health will face 21.6 million patients a year by 2030 [3]. The increasing trend due to cancer will pose a threat to human development, necessitating developing action plans and political commitments to improve cancer control.

Quantitative assessment of the epidemiological measures on cancer would be mandatory for developing effective cancer control plans [4]. Providing such measures are more critical for developing countries like Iran that are facing many challenges in cancer management, such as rapid socioeconomic development and a growing and aging population [5–7]. Epidemiological measures on cancer are best provided by cancer registries; however, the pace of development of cancer registries is slow, and high-quality cancer data is not adequately available in many developing countries due to lack of human and financial resources [5]. A national pathology-based cancer registry program has been launched in Iran since 2000 by the ministry of health, covering all the 31 provinces of the country [8]. Although the overall completeness of the Iran cancer registry program is estimated between 60 and 75 percent, which is not ideal, it is a guide for Iranian policymakers to make more informed decisions about cancer control [7, 9–11].

Cancer incidence is an important epidemiological measure that could be used to estimate the overall effectiveness of health programs and required investment for cancer control. However, there is only a little published information regarding cancer incidence in Iran [7, 12]. The objective of this study is to present the cancer incidence of specified 22 cancer groups in Iran in all 31 provinces of the country from 2000 to 2016 for both sexes and across all age groups. Herein, we reanalyzed the existing data of the cancer registration program of Iran and implemented data presentation methods used by the Global Burden of Disease (GBD) [2, 4, 13].

## 2. Material and Methods

### 2.1. Data source

To study Iran's national and provincial cancer incidence, we extracted data from the Cancer Project, which collects the Iranian cancer registry data and visualizes it in the VIZIT data visualization system [14]. The national cancer registry data is collected from pathology centers, hospitals, and death registries [15]. The main data that is obtained from each cancer case include identification characteristics, age, gender, place of residence, codes of diagnosed cancer, and year of initial diagnosis [15]. Since the first available published report of the cancer registry in Iran dates back to 2000 [16], we limited the study period to the years 2000 to 2016.

The number of cancer incidence cases at the global level and in each sociodemographic index (SDI) quintile was extracted from the supplemental content of the 2016 Global Burden of Disease Study [2]. SDI is a composite measure of development status, which is strongly correlated with health outcomes. The index is a geometric mean of the income per capita, average educational attainment, and fertility rates of specified regions [17].

### 2.2. Definition

Classification of the cancer types was defined by the International Statistical Classification of Diseases and Related Health Problem 10th Revision (ICD-10). Twenty-two cancer groups were included in the study, and eTable 1 represents specified ICD-10 codes allocated to each group. The classification of cancer groups in VIZIT and the 2016 GBD study was matched with the defined cancer groups of our study.

### 2.3. Statistical analysis

The methodology and statistical analysis that is used in this study for calculating cancer incidence, crude rate (CR), and age-standardized rate (ASR) follow the cancer project study protocol [18, 19]. This project was designed to estimate cancer incidence in Iran and provides data on cancer incidence, crude rate, and age-standardized rate for 70 subtypes of cancer in different age groups from 1990 to 2016 [19]. For calculating the age-standardized rate, the direct method of standardization was implemented, and the population of Iran in 2016 was used as the standard population. Joinpoint analysis was performed using Joinpoint Trend Analysis Software (Version 4.9.0.0) to calculate the average annual percent change of crude rate and age-standardized rate for each cancer group from 2000 to 2016.

## 3. Results

### 3.1. National Cancer Incidence

The incidence of cancer was 126,982 patients, and the crude rate (CR) for both sexes was 155 per 100,000 people in 2016 (Table 1). The most common cancer groups in both sexes combined were breast (14568 cases), skin (11712 cases), and colorectal cancers (11422 cases) (Table 1). In women, breast cancer was by far the most common cancer (14217 cases) and accounted for 22% of all cancer cases. Colorectal (5717 cases) and skin cancers (5354 cases) were the second and third most common cancers in women, respectively. In men, the most common cancers were prostate (7963 cases), skin (6357 cases), and stomach cancers (5477 cases), which altogether accounted for 30% of all cancer cases.

**Figure 1:**
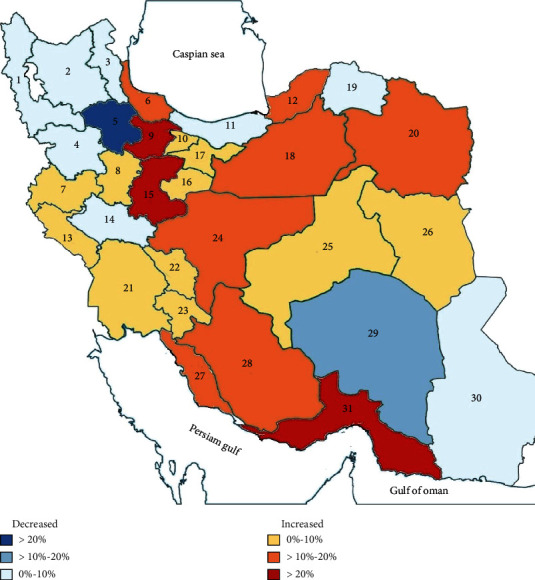
Relative changes in cancer age-standardized rate for both sexes from 2000 to 2016 in provinces of Iran. 1 indicates West Azarbayjan, 2: East Azarbayjan, 3: Ardabil, 4: Kurdistan, 5: Zanjan, 6: Gilan, 7: Kermanshah, 8: Hamadan, 9: Qazvin, 10: Alborz, 11: Mazandaran, 12: Golestan, 13: Ilam, 14: Lorestan, 15: Markazi, 16: Qom, 17: Tehran, 18: Semnan, 19: North Khorasan, 20: Khorasan Razavi, 21: Khuzestan, 22: Chaharmahal and Bakhtiari, 23: Kohkiluye and Bouyerahmad, 24:Isfahan, 25: Yazd, 26: South Khorasan, 27: Boushehr, 28: Fars, 29: Kerman, 30: Sistan and Balouchestan, and 31: Hormozgan.

Cancer incidence approximately doubled between 2000 and 2016, and an increase in the number of incident cases occurred in all cancer groups except esophageal cancer. The average annual percent change of the crude rate (CR) was significantly positive in both sexes for 14 out of 21 specified cancer groups. Stomach cancer was the only cancer with a different trend of CR in women and men, which was increasing for women but decreasing in men (Table 1). The annual percent change in both CR and ASR was higher in women than men (Tables 1 and 2). The age-standardized rate (ASR) also increased from 146.4 per 100,000 in 2000 to 155 per 100,000 in 2016. The ASR of cancer in women had an increasing trend in 26 provinces, while in men, only 16 provinces showed an increased ASR (Table 2). Markazi, Hormozgan, and Qazvin showed the highest annual percent change of ASR for women among all provinces. For men, Markazi, Bushehr, and Hormozgan showed the highest ASR annual percent change. Zanjan, Kerman, and Lorestan were the only provinces with a decreasing trend in cancer ASR for both women and men (Table 2, Figure 1).

**Figure 2:**
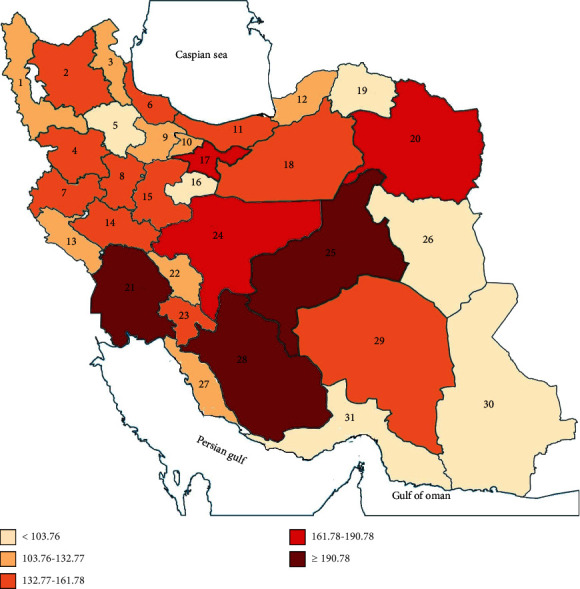
Cancer age-standardized rate (ASR) for both sexes in provinces of Iran in 2016. 1 indicates West Azarbayjan, 2: East Azarbayjan, 3: Ardabil, 4: Kurdistan, 5: Zanjan, 6: Gilan, 7: Kermanshah, 8: Hamadan, 9: Qazvin, 10: Alborz, 11: Mazandaran, 12: Golestan, 13: Ilam, 14: Lorestan, 15: Markazi, 16: Qom, 17: Tehran, 18: Semnan, 19: North Khorasan, 20: Khorasan Razavi, 21: Khuzestan, 22: Chaharmahal and Bakhtiari, 23: Kohkiluye and Bouyerahmad, 24:Isfahan, 25: Yazd, 26: South Khorasan, 27: Boushehr, 28: Fars, 29: Kerman, 30: Sistan and Balouchestan, and 31: Hormozgan.

Cancer incidence showed a heterogeneous distribution in Iran. For instance, cancer ASR varied from 219.7 in Khuzestan to 74.7 in Sistan and Balouchestan in 2016 (Figure 2, Table 2). The seven provinces that their cancer ASR was higher than the national average were Khuzestan, Yazd, Fars, Tehran, Khorasan Razavi, Isfahan, and Semnan (Table 2).

**Figure 3:**
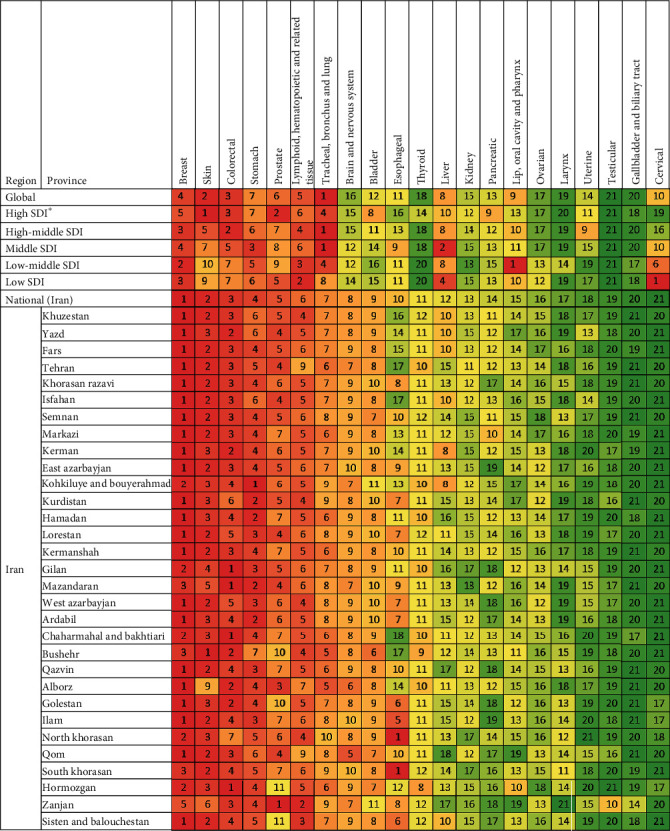
Cancers ranked by number of incident cases in both sexes, globally, by development status, nationally, and provincially in 2016. Colors correspond to the ranking (dark red is the cancer group with the most incidence, and dark green is the cancer group with the least incidence for the location indicated). The numbers inside each box indicate the ranking. The cancers were sorted by their ranking in national incidence. “Other cancers” group is not included in the ranking.∗SDI: social development index.

The ranking of cancer groups by incident cases was different between the global and national levels (Figure 3). For instance, the “trachea, bronchus, and lung” cancer group has the highest incidence worldwide; however, it was ranked seventh in Iran. As another example, cervical cancer was the eleventh most incident cancer worldwide; however, it was the least incident cancer group in Iran. Likewise, the incidence of cancer groups in Iran was also heterogeneous. For instance, esophageal cancer was ranked tenth in Iran, but it was the most incident cancer in the North and South Khorasan. Despite such differences, the seven most common cancers in Iran were similar to the seven most common cancers globally, including breast, skin, colorectal, stomach, prostate, hematopoietic, and lung cancers.

The rank of cancer groups by incidence was different between different SDI quintiles. For example, cervical cancer had the highest incidence in low-SDI countries; however, it is nineteenth cancer by incidence in high-SDI countries. The pattern of cancer groups' ranking by the number of incident cases in Iran, as a high-middle SDI country [20], was similar to high-middle and high-SDI countries (Figure 3).

**Figure 4:**
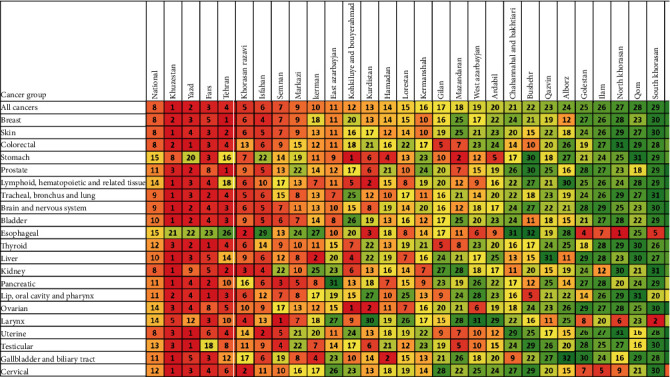
Iran's provinces ranked by age-standardized rate (ASR) in both sexes for each cancer group^1^, 2016. ^1^National ASR is also included in the first column for better comparison. Colors correspond to the ranking (dark red is the cancer group with the most incidence, and dark green is the cancer group with the least incidence for cancer indicated). The numbers inside each box indicate the ranking. The provinces were sorted based on their 2016 ASR in Iran. “Other cancers” group is not included in the ranking.

The provinces were ranked by their age-standardized rate (ASR) for each cancer group. This ranking enables comparing the rank of each province in the ASR of a specified cancer group with the rank of the province in the ASR of all cancers combined to find discrepancies (Figure 4). The comparison suggests that the distribution of some cancer groups does not follow the general distribution of cancer in Iran. For example, the fourth province in prostate cancer ASR and the second province in testicular cancer ASR was Zanjan which is the thirtieth province in cancer ASR. This indicates a surprisingly high incidence of prostate and testicular cancer in Zanjan. Similar examples are the high ASR of stomach cancer in Kohkiluyeh and Bouyerahmad, Mazandaran, Hamadan, and Ardabil; the high ASR of esophageal cancer in North, Razavi, and South Khorasan, Golestan, and Kurdistan; the high ASR of “Lip, oral cavity, and pharynx” cancer in Bushehr, and so forth.

### 3.2. The Pattern of Cancer Incidence by the Age Group

**Figure 5:**
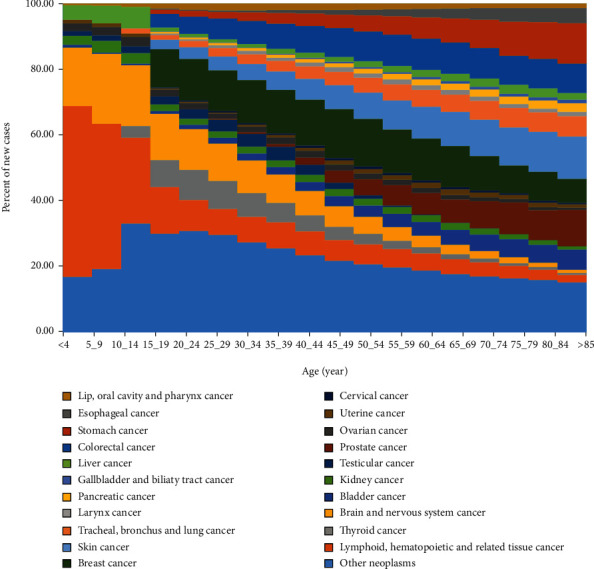
Age-specific contributions of cancer types to total cancer incidence for both sexes, 2016. For International Classification of Disease codes pertaining to each neoplasm group, see eTbale 1 in the supplement.

For childhood cancers (age<14 years), the three most common cancers excluding “other neoplasms” were lymphoid, hematopoietic, and related tissue; brain and nervous system; and liver cancers. In young- and middle-aged adults (age 15-54 years), the cancer groups that had the highest incidence were breast, colorectal, and brain and nervous system cancers. For the population older than 54 years, skin, breast, and colorectal cancers were the cancer groups with the highest number of incident cases (Figure 5).

### 3.3. The Cancers with the Highest Incidence in Iran in 2000 and 2016

**Figure 6:**
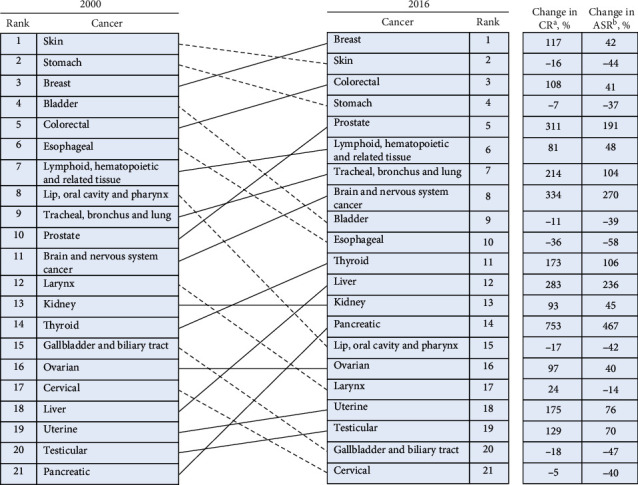
Cancers ranked for both sexes by national crude rate, including the percentage change in crude rate and the percentage change in the age-standardized rate between 2000 and 2016. The “other cancer” group is not included here because it contains multiple different types of cancers. Solid lines connecting the 2000 and 2016 charts indicate increased or unchanged rank for the connected cancers; dotted lines indicated decreased rank. ^a^CR: crude rate; ^b^ASR, age-standardized rate.

The cancer groups with the highest crude rate in 2000 were skin, stomach, and breast cancer, whereas the cancer groups with the highest crude rate in 2016 were breast, skin, and colorectal cancer (Figure 6). Pancreatic, brain, and nervous system and liver cancers were the cancer groups that their ASR increased more than two times from 2000 to 2016. On the other side, esophageal; gallbladder and biliary tract; skin; lip, oral cavity, and pharynx; cervical; bladder; and stomach cancers were the cancer groups that their ASR decreased from 2000 to 2016 (Figure 6).

## 4. Discussion

We found that cancer incidence, crude rate, and age-standardized rate (ASR) increased in Iran from 2000 to 2016 with vast heterogeneity by cancer types, provinces, and sexes. Cancer incidence increased by 97% from 2000 to 2016 (Tables 1 and 2). Such an increase could be possibly due to the growth and aging of the population, improvements in the data recording system, and the increase in the prevalence of cancer risk factors [21–24]. Comparing percentage increases in incidence, crude rate, and ASR from 2000 and 2016 reveals that population growth accounts for 31%, and population aging accounts for 55% out of 97% increase in cancer incidence. Therefore, the remaining 11% could be attributed to other causes such as improvements in the data recording system and the increase in the prevalence of cancer risk factors. Notably, this attribution was calculated according to all cancer types' incidence and could not be generalized to each defined cancer group. Considering the projected aging of Iran's population in the following years, a further increase in cancer incidence is expected, which would be a significant health concern [25, 26].

The study estimates that the cancer ASR using the world standard population was 147.9 for women and 159.5 per 100,000 people for men in Iran in 2016. This ASR is lesser than the global cancer ASR estimated by the GBD study in 2016 (213.9 per 100,000 people for women and 306.8 per 100,000 people for men) [2] and the GLOBOCAN study in 2018 (182.6 per 100,000 people for women and 218.6 for men) [27]. The difference in ASR estimates could be due to the incompleteness of the cancer registry program in Iran [9, 11]. Upcoming sections will discuss study results for some cancer groups.

### 4.1. Breast Cancer

It was shown that breast cancer had the highest incidence among cancer groups in Iran and most of its provinces, which was in accordance with some previous studies [28]. However, breast cancer is not the most common cancer in the world or any SDI quintiles [2, 27]. This difference indicates a disproportionate high incidence of breast cancer in Iran.

### 4.2. Skin Cancer

Skin cancer (including melanoma and non-melanoma skin cancers) ranked second by incidence in Iran. Besides, this cancer was the most common cancer type in the older adults and elderly (age > 54 years) (Figure 5). Although a previous study introduced Qom as a hot spot for skin cancer in 2009, our results showed a low level of skin cancer ASR in this province [29]. Moreover, it was shown that the ranking of provinces by skin cancer ASR was similar to the pattern of cancer ASR in Iran (Figure 4).

### 4.3. Colorectal Cancer

Colorectal cancer had the third highest ASR in 2016, which shows a 41% increase since 2000. Such an increase in colorectal cancer incidence could be partly due to changes in the dietary habits of the Iranian population, lower consumption of vegetables and fruits and higher intake of red meat, and the community transition to a sedentary lifestyle [24, 30]. Furthermore, it was surprisingly shown that colorectal cancer incidence was higher in women than men in 2016. This higher incidence shows a sex transition in the incidence pattern of this cancer in Iran [24, 31]. We found that Mazandaran, Gilan, and Boushehr had high ASR in colorectal cancer in proportion to their all cancer types' ASR (Figures 3 and 4). The unexpected high ASR of colorectal cancer in Gilan and Mazandaran ws known before [30]; however, most of the previous studies did not mention Boushehr as a province with unexpected high colorectal cancer ASR. High prevalence of obesity and inflammatory bowel disease (IBD) in Mazandaran and Gilan and low physical activity in Boushehr might be the risk factors for the mentioned unexpected high ASR of colorectal cancer in these provinces [32–35].

### 4.4. Stomach Cancer

Stomach cancer was the fourth cancer by incidence in 2016. It was shown that there is a disproportionate high ASR of this cancer in the western and northern provinces of Iran (Kohkiluye and Bouyerahmad, Kurdistan, and Hamadan, Gilan, Mazandaran, and Ardabil) (Figure 4). Previous studies also determined a similar epidemiologic distribution [36, 37]; however, they did not recognize Kohkiluye and Bouyerahamd as the province with the highest ASR for stomach cancer. A combination of H. pylori infection, gastroesophageal reflux disease, smoking, and heavy metals was reported as risk factors of gastric cancer in these provinces [38–40].

### 4.5. Prostate Cancer

Prostate cancer was the tenth and fifth cancer by incidence in 2000 and 2016, respectively. The cancer ASR increased by 191% during this period (Figure 6). Such a sharp increase could be due to the urbanization of the country, which is accompanied with increasing high-fat diet consumption, decreasing energy expenditure and physical activity, higher levels of pollution, more contact with carcinogens, and higher cancer detection rates [41, 42]. It was shown that Tehran, Yazd, and Khuzestan were the provinces with the highest prostate cancer ASR. Besides, Zanjan, Kurdistan, and Mazandaran showed high ASR for prostate cancer in comparison with their all cancer types' ASR (Figure 4). Accordingly, a previous study demonstrated that the relative risk for prostate cancer after adjusting its risk factors was much higher in Zanjan, Kurdistan, and Mazandaran provinces than the average national level [42].

### 4.6. Esophageal Cancer

This study, following some other studies, showed that esophageal cancer incidence is decreasing in Iran [41, 43]. This cancer was the tenth cancer by incidence in 2016 and was the first cancer by incidence in South and North Khorasan (Figure 3). North, Razavi, and South Khorasan, Golestan, and Kurdistan were the provinces with the highest ASR for this cancer (Figure 4). These results approved previous reports, indicating that these provinces have one of the highest reported rates of esophageal squamous cell carcinoma worldwide. Most of the aforementioned provinces have common borders with Turkmenistan and Afghanistan countries that have high ASR for esophageal cancer due to their susceptible genetic/ethnic background [44–46]. Besides genetic susceptibility, exposure to polycyclic aromatic hydrocarbons (from opium smoking and indoor air pollution), drinking hot tea, low intake of fruits and vegetables, and drinking unpiped water were determined as risk factors for esophageal cancer in these areas [47, 48].

### 4.7. Tracheal, Bronchus, and Lung Cancer

The tracheal, bronchus, and lung cancer group had the highest cancer incidence worldwide in 2016; however, it was the seventh cancer by incidence in Iran. Therefore, this cancer showed one of the most prominent differences in ranking of cancer types by incidence between Iran and other countries of the world (Figure 3). It was shown that the ranking of provinces by this cancer ASR was majorly similar to the pattern of cancer ASR in Iran, having the highest ASRs in Khuzestan, Yazd, and Fars (Figure 4). Surprisingly, these provinces were not the highest in cigarette and water-pipe smoking prevalence among provinces of Iran [49]. However, these provinces, especially Khuzestan and Fars, along with other provinces with high ASRs like Tehran, Isfahan, and Khorasan Razavi, had the highest premature deaths attributable to ambient particulate matter 2.5 in 2016 [50]. Provinces like West and East Azarbayjan, Hamadan, and Boushehr had higher ranks in the tracheal, bronchus, and lung cancer ASR in comparison with their ranks in all cancers ASR, which could be attributed to the high prevalence of smoking and high concentration of air pollutions [50, 51] (Figure 4).

### 4.8. Limitations

We faced some limitations in defining cancer groups due to the lack of data for some subgroups. For example, Hodgkin lymphoma, non-Hodgkin lymphoma, leukemia, and multiple myeloma had not been specified in the data. Therefore, the “hematopoietic, lymphoid, and related tissue” group was defined, which includes all the four mentioned cancer subgroups. Another example was melanoma and nonmelanoma skin cancers, and the “skin cancer” group was defined to cover both subgroups.

## 5. Conclusion

We showed that cancer incidence, crude rate, and age-standardized rate (ASR) in Iran increased from 2000 to 2016 with vast heterogeneity by cancer type, province, and sex. It was also shown that the crude rate of cancer in Iran was 155 per 100,000 people for both sexes combined in 2016, much less than the global cancer crude rate estimated by the GBD study in 2016 and the GLOBOCAN study in 2018. This study represents quantitative data on different cancer types' incidence in Iran's provinces, which would help the appropriate allocation of resources to each part of the health care system and the development of effective national cancer control plans [4, 13].

## Figures and Tables

**Table 1:** National incidence, crude rate, and average annual percent change of the crude rate for all cancers and 22 cancer groups in 2000-2016^a,b^.

Cancer group	Incident cases, 2000, national		Crude rate (per 100 000), national, 2000		Incident cases, 2016, national		Crude rate (per 100 000), national, 2016		Average annual percent change of crude rate, 2000-2016 (95% CI)	
	Female	Male	Female	Male	Female	Male	Female	Male	Female	Male
All cancers	28288.3	36212.6	91.69	113.66	62881.8	64099.9	153.8	156.24	3.3 (3.2, 3.3)	2 (2, 2.1)
Breast	4991.3	140.6	16.18	0.44	14217.1	351.2	34.77	0.86	4.9 (4.8, 5)	4.3 (4, 4.5)
Skin	4310.3	6359.4	13.97	19.96	5354.9	6357.6	13.1	15.5	-0.4 (-0.5, -0.3)	-1.6 (-1.7, -1.5)
Colorectal	1876.1	2135.4	6.07	6.83	5717.6	5298	13.99	12.92	5.3 (5.2, 5.5)	4.1 (3.9, 4.2)
Stomach	2402.6	4834.8	7.79	15.17	3327	5477.1	8.14	13.35	0.3 (0.1, 0.4)	-0.8 (-1, -0.6)
Prostate	…	1478.3	…	4.64	…	7963.7	...	19.41	…	9.4 (9.2, 9.5)
Lymphoid, hematopoietic, and related tissue	1118.9	1935.8	3.63	6.08	2976.6	4269.7	7.28	10.41	4.3 (3.7, 4.8)	3.4 (3.3, 3.5)
Tracheal, bronchus, and lung	506.4	1089.2	1.64	3.42	2208.6	4015	5.4	9.79	7.7 (7.5, 7.9)	6.8 (6.7, 6.9)
Brain and nervous system	357	626	1.16	1.97	2263.3	3620	5.39	8.25	10.1 (9.9, 10.3)	9.4 (9.2, 9.6)
Bladder	978.3	3525.4	3.17	11.07	1216.4	4028	2.98	9.82	-0.4 (-0.5, -0.3)	-0.7 (-0.8, -0.7)
Esophageal	1992.1	2047.8	6.46	6.43	1984.7	1431.8	4.85	3.49	-1.8 (-1.9, -1.7)	-3.8 (-3.9, -3.6)
Thyroid	627.2	294.8	2.03	0.93	2327.3	968.2	5.69	2.36	6.6 (6.4, 6.9)	6 (5.7, 6.3)
Liver	217.2	315.5	0.7	0.99	1228.8	1444.3	3.01	3.52	9.5 (9.4, 9.7)	8.2 (8.1, 8.4)
Kidney	354.6	597	1.15	1.88	961.7	1447.7	2.35	3.53	4.6 (4.4, 4.7)	4 (3.9, 4.2)
Pancreatic	88.6	114	0.29	0.36	1108.9	1123.1	2.71	2.74	15 (14.6, 15.4)	13.6 (13.3, 13.8)
Lip, oral cavity and pharynx	730.7	1076.2	2.37	3.38	883.8	1092.5	2.16	2.66	-0.6 (-0.8, -0.3)	-1.5 (-1.6, -1.4)
Ovarian	725	…	2.35	…	1875.8	...	4.59	...	4.2 (4.1, 4.4)	…
Larynx	150.6	825.9	0.49	2.59	266	1321.1	0.65	3.22	1.8 (1.5, 2)	1.4 (1.2, 1.5)
Uterine	429	…	1.39	…	1535.1	...	3.75	...	9.8 (9.1, 10.5)	…
Testicular	…	388.6	…	1.22	…	1160.4	...	2.83	…	5.4 (5.2, 5.6)
Gallbladder and biliary tract	508	261.7	1.65	0.82	517.7	319.8	1.27	0.78	-4.3 (-4.4, -4.1)	-5.9 (-6.2, -5.6)
Cervical	638.3	…	2.07	…	791.6	...	1.94	...	-0.4 (-0.6, -0.2)	…
Other neoplasms	5286.1	8165.3	17.13	25.42	12118.1	12409.9	29.78	30.8	3.8 (3.4, 4.1)	1.2 (0.9, 1.5)

^a^Cancer groups are defined based on International Statistical Classification of Diseases and Related Health Problems, Tenth Revision (ICD-10) codes. These codes have been shown in detail in eTable 1 in supplements. ^b^Cancers have been sorted based on the crude rate for both women and men combined in 2016.

**Table 2:** National cancer incidence, age-standardized rate, and average annual percent change of age-standardized rate in 2000-2016 among provinces of Iran^a^.

Provinces	Incident cases, 2000		Age-standardized rate (per 100 000), 2000		Incident cases, 2016		Age-standardized rate (per 100 000), 2016		Average annual percent change of age-standardized rate, 2000-2016 (95% CI)	
	Female	Male	Female	Male	Female	Male	Female	Male	Female	Male
Khuzestan	2032.9	2613	192.8	220.5	4460.8	4321.8	221.4	218.1	0.8 (0.7, 0.8)	0.0 (0.0, 0.0)
Yazd	547.4	707.3	177.4	208.1	1193.3	1364.2	197.8	199.8	0.7 (0.7, 0.8)	-0.3 (-0.4, -0.3)
Fars	2108.3	2660.3	167.2	187.4	5077.2	4784.7	199.9	190.9	0.8 (0.8, 0.8)	0.4 (0.4, 0.4)
Tehran	5609.5	7180.6	167.1	186.8	13693	13242.4	192.5	181.1	0.5 (0.5, 0.5)	0.2 (0.1, 0.2)
Khorasan Razavi	2362.2	3050.3	146.2	167.3	5442.3	5348.9	176.3	169.5	0.9 (0.9, 0.9)	0.3 (0.3, 0.4)
Isfahan	1952.1	2453.6	133.4	148.6	5001.5	4883.9	169.6	167.7	1.4 (1.4, 1.5)	0.8 (0.8, 0.8)
Semnan	266.6	340.8	132.9	152.8	602.4	593.9	160.7	158.5	1.1 (1.1, 1.1)	0.3 (0.3, 0.4)
National	28288.3	36212.5	137.7	155.1	62881.7	64099.8	153.8	156.2	0.7 (0.7, 0.7)	0.0 (0.0, 0.1)
Markazi	505.5	624.1	105.0	118.7	1312	1291.5	149.8	150.5	2.2 (2.2, 2.3)	1.5 (1.5, 1.5)
Kerman	1031.1	1374.9	153.5	179.3	2235.1	2162	152.6	145.8	-0.3 (-0.4, -0.3)	-1 (-1, -1)
East Azarbayjan	1703.0	2152.5	145.5	160	3179.1	3221.2	148.8	149.1	0.3 (0.3, 0.3)	-0.5 (-0.5, -0.4)
Kohkiluye and Bouyerahmad	198.5	254.5	131.7	149.3	508.2	454.4	145	150.4	0.8 (0.8, 0.9)	-0.2 (-0.2, -0.2)
Kurdistan	633.6	861.6	151	170.1	1147.4	1150.8	144.1	150.5	-0.0 (-0.1, 0.0) †	-1 (-1, -1)
Hamadan	718.5	917.8	123.5	141.4	1494.1	1496.1	144.4	145.7	1 (1, 1.1)	0.1 (0.1, 0.2)
Lorestan	679.7	898.9	147	163.7	1325.1	1290.4	143.5	143.4	-0.2 (-0.2, -0.1)	-0.8 (-0.8, -0.8)
Kermanshah	687.0	922.4	125.2	139.7	1471.4	1503.6	138.6	141.3	0.8 (0.7, 0.8)	-0.1 (-0.1, -0.0)
Gilan	1124.1	1321.9	119.7	134	2300	2394.9	138.6	140.5	1 (1, 1)	0.2 (0.2, 0.2)
Mazandaran	1340.9	1637	134.2	151.5	2555.8	2774.3	134.8	144.2	0.4 (0.4, 0.5)	-0.7 (-0.7, -0.7)
West Azarbayjan	1023.2	1274.6	126.1	138.8	1965.4	2233.6	126.7	137.1	0.5 (0.5, 0.5)	-0.6 (-0.6, -0.6)
Ardabil	447.9	578	122.1	139.6	827.7	841.3	126.6	130.3	0.4 (0.4, 0.4)	-0.6 (-0.6, -0.6)
Chaharmahal and Bakhtiari	268.7	345.1	114.9	129.3	570.5	546.2	125.7	121.6	0.4 (0.3, 0.4)	-0.2 (-0.2, -0.1)
Bushehr	218	268.4	96.3	106	774.1	531.1	127.3	115	1.1 (1.1, 1.1)	1.2 (1.1, 1.2)
Qazvin	295.7	380	90.1	102.2	788.8	752.6	120.2	116.6	1.6 (1.6, 1.7)	1 (1, 1)
Alborz	518.8	636.7	107.3	110.7	1639.1	1461.5	121.7	114.5	0.4 (0.4, 0.4)	0.6 (0.6, 0.6)
Golestan	403.9	562.6	92.4	116.8	1028.2	953.4	122.8	113.1	1.3 (1.2, 1.3)	0.3 (0.3, 0.4)
Ilam	127.0	182.7	93.3	108.6	322.3	284.5	108.9	106.7	0.8 (0.8, 0.9)	0.0 (0.0, 0.0)
North Khorasan	231.1	289.3	101.4	113.4	464.5	442.7	105.8	101.3	-0.0 (-0.0, 0.0) †	-0.4 (-0.4, -0.4)
Qom	237.3	299.4	85.5	92.9	549.8	515.3	97.6	96.9	0.8 (0.8, 0.8)	0.3 (0.3, 0.3)
South Khorasan	186.7	245.3	86.6	103.6	366.8	338.2	99.8	91.3	0.3 (0.3, 0.3)	-0.2 (-0.3, -0.2)
Hormozgan	215.4	299.7	66.1	78.2	668.3	605.4	92.6	86.1	1.7 (1.6, 1.7)	1.1 (1, 1.1)
Zanjan	294.3	382.3	98.5	113.9	453.9	433.5	81.7	80	-1.3 (-1.3, -1.3)	-2 (-2.1, -2)
Sistan and Balouchestan	318	465.8	70	82.9	680.2	661.8	75.4	74	0.3 (0.3, 0.4)	-0.6 (-0.6, -0.5)

^a^Provinces have been sorted based on the age-standardized rate for both women and men combined in 2016. †nonsignificant change.

## Data Availability

The datasets used and/or analyzed during the current study are available from the corresponding author on reasonable request. Besides, data are available from https://vizit.report/en/dashboard.html

## Supplementary Materials

Supplementary Materials eTable 1: list of International Classification of Disease (ICD) codes mapped to the article cause list for cancer.
