# Supplementary material for: National and Subnational Cancer Incidence for 22 Cancer Groups, 2000 to 2016: A Study Based on Cancer Registration Data of Iran
Source: J Cancer Epidemiol. 2021 Jul 12;2021:6676666. doi: 10.1155/2021/6676666 (PMC8292090; doi:10.1155/2021/6676666)
Supplement: Supplementary Materials — eTable 1: list of International Classification of Disease (ICD) codes mapped to the article cause list for cancer. [file 6676666.f1.docx]

| **eTable 1: List of International Classification of Disease (ICD) codes mapped to the article cause list for cancer** | |
| --- | --- |
| **Cause** | **ICD-10 Code** |
| All cancers | C00-97 |
| Lip, oral cavity and pharynx | C00-C14 |
| Esophageal | C15 |
| Stomach | C16 |
| Colorectal cancer | C18-21 |
| Liver | C22 |
| Gallbladder and biliary tract | C23-24 |
| Pancreatic | C25 |
| Larynx | C32 |
| Trachea, bronchus, and lung | C33-34 |
| Skin | C43-C44 |
| Breast | C50 |
| Cervical | C53 |
| Uterine | C54 |
| Ovarian | C56 |
| Prostate | C61 |
| Testicular | C62 |
| Kidney | C64-65 |
| Bladder | C67 |
| Brain and nervous system | C70-72 |
| Thyroid | C73 |
| Lymphoid, hematopoietic and related tissue cancer | C81-96 |
| Other | C17,C26,C30-31,C37-39,C40-41,C45-49,C51-52,C55.C57-58,C60,C63,C66,C68,C69,C74-75,C76-80,C97 |
